# Dementia remains the major predictor of death among octogenarians. A study of two population cohorts of 85-year-olds examined 22 years apart

**DOI:** 10.1007/s10654-021-00745-5

**Published:** 2021-04-21

**Authors:** Hanna Wetterberg, Jenna Najar, Lina Rydén, Mats Ribbe, Therese Rydberg Sterner, Anna Zettergren, Xinxin Guo, Hanna Falk Erhag, Simona Sacuiu, Silke Kern, Ingmar Skoog

**Affiliations:** 1 Neuropsychiatric Epidemiology Unit, Institute of Neuroscience and Physiology, Department of Psychiatry and Neurochemistry, Centre for Ageing and Health (AGECAP), Sahlgrenska Academy, University of Gothenburg, Wallinsgatan 6, 431 41 Mölndal, SE Sweden; 2 Department of Psychiatry Cognition and Old Age Psychiatry, Region Västra Götaland, Sahlgrenska University Hospital, Gothenburg, Sweden

**Keywords:** Epidemiology, Dementia, Life expectancy, Mortality, Secular trends, Cohort studies

## Abstract

Dementia is the major predictor of death in old age. The aim of this paper was to determine whether 8-year mortality among 85-year olds with and without dementia, and if the contribution of dementia to mortality relative to other common diseases has changed. We used two population-based cohorts of 85-year-olds (N = 1065), born in 1901–02 and 1923–24, which were examined with identical methods in 1986–87 and 2008–2010 and followed for 8-year mortality according to data from the Swedish Tax Agency. Dementia was diagnosed according to DSM-III-R. Other diseases were diagnosed based on self-reports, close informant interviews, somatic examinations, and the Swedish National In-patient Register. Compared to cohort 1901–02, cohort 1923–24 had a lower 8-year mortality both among those with (HR 0.7; 95% CI 0.5–0.99) and without dementia (HR 0.7; 95% CI 0.5–0.9). Dementia was associated with increased mortality in both cohorts (cohort 1901–02, HR 2.6; 95% CI 2.0–3.2, cohort 1923–24, HR 2.8; 95% CI 2.3–3.5), and remained the major predictor of death, with a population attributable risk of 31.7% in 1986–87 and 27.7% in 2008–10. Dementia remained the most important predictor of death in both cohorts. The relative risk for mortality with dementia did not change between cohorts, despite a decreased mortality rate in the population.

**Supplementary Information:**

The online version contains supplementary material available at 10.1007/s10654-021-00745-5.

## Introduction

The increased life expectancy worldwide will result in a substantial increase in the number of individuals living with dementia, from almost 50 million today to 132 million by 2050 [1]. Dementia is one of the strongest predictors of mortality among older adults [2–4]. A number of recent studies suggest that the age-specific incidence of dementia declines in Western countries [5–10]. It is not clear whether the contribution of dementia on mortality has changed when both life expectancy increases and dementia incidence decreases. Furthermore, whether the declining incidence of dementia will result in a lower age-specific prevalence of dementia partly depends on whether mortality increases or decreases to a similar extent among individuals with and without dementia [11]. Factors found to predict a shorter length of survival in dementia are high age, male sex, and severity of the disease [12], but also midlife sociodemographic factors and cardiovascular risk factors [13]. As the frequency of the latter factors have changed [14–16], survival in dementia might also change.

Few studies have investigated time-trends in mortality in relation to dementia, showing conflicting results [17–21]. Studies investigating time-trends in mortality among populations above age 80 (i.e. the age group expected to grow the most and which includes most cases of dementia), are scarce [11]. We have previously reported that dementia is a major cause of mortality among 85-year-olds born 1901–02 [4]. We have now examined a new cohort of 85-year-olds born 22 years later. The aim of this study was to examine whether 8-year mortality in relation to dementia among 85-year olds has changed. In addition, we examined the importance of dementia relative to other common diseases to predict mortality in the two cohorts.

## Methods

### Study population

**Fig. 1:**
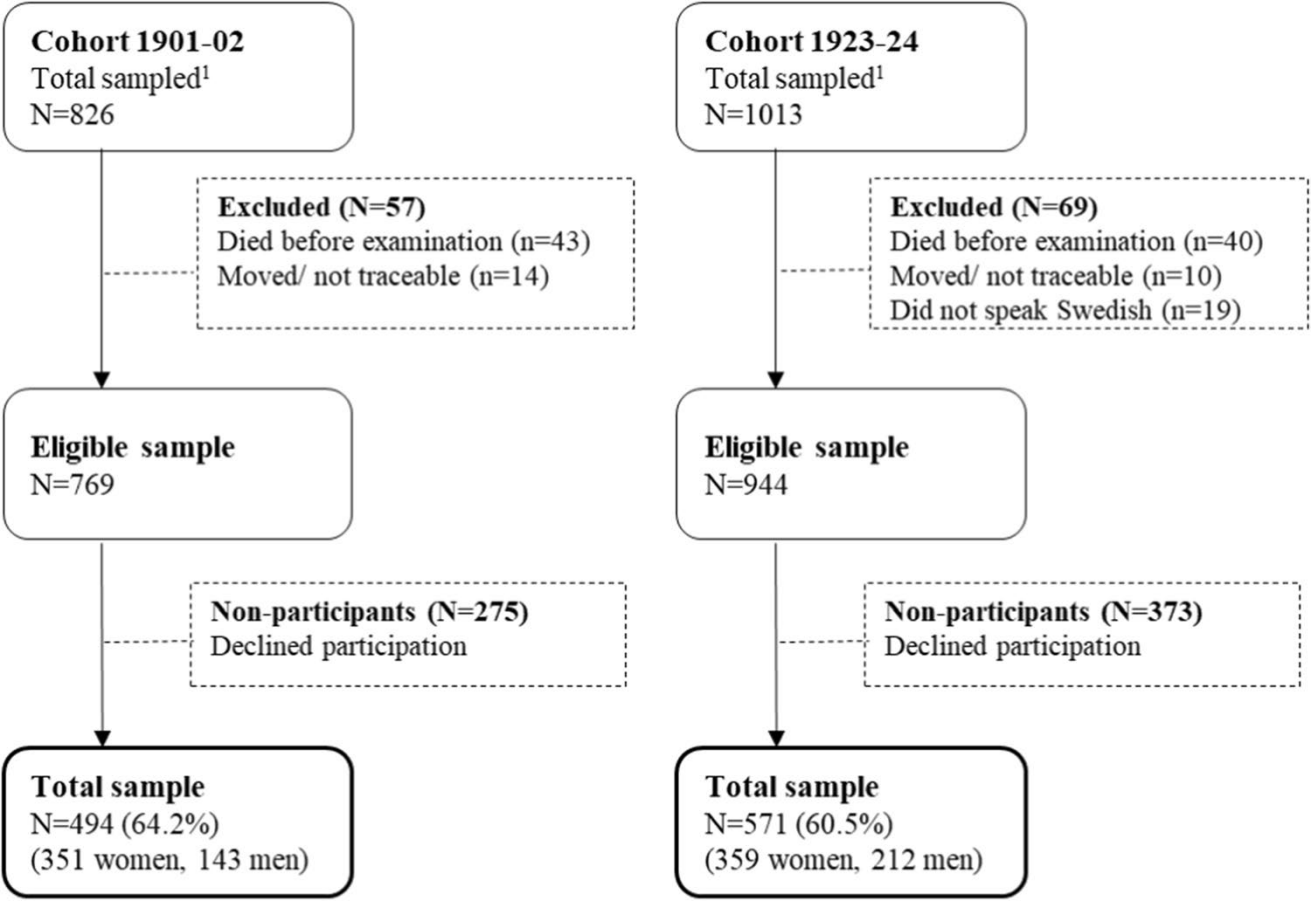
Sample flow-chart of cohort 1901–02 and 1923–24. In cohort 1901–02, every second 85-year-old in Gothenburg, Sweden, born July 1^st^, 1901 to June 30^th^, 1902 were invited to the examination in 1986–87 (n = 826). Forty-three individuals died before the examination, and 14 had moved or could not be traced, leaving an eligible sample of 769, of which 494 participated (response rate 64.2%; 143 men and 351 women). In cohort 1923–24, every second 85-year-old in Gothenburg, Sweden, born July 1st, 1923, to June 30th, 1924, were invited to the examination in 2008–2010 (N = 1013). Forty individuals died before the examination, 19 could not speak Swedish, four had emigrated outside Sweden and six could not be traced, leaving an eligible sample of 944 individuals, of which 571 (response rate 60.5%; 212 men and 359 women) participated

Two cohorts of 85-year-olds from the Gothenburg H70 Birth Cohort studies, Sweden, born 1901–02 and 1923–24, were examined in 1986–87 (N = 494; response rate 64.2%) and 2008–10 (N = 571, response rate 60.5%) (Fig. 1). Both samples were systematically obtained from the Swedish Population Register based on birth dates, and included persons living in private households and in institutions. In cohort 1901–02, non-participants and participants were similar regarding sex (women 71% versus 75%), 3-year mortality rate (28.7% versus 25.9%) [22], and National Inpatient Register diagnoses for cardiovascular disorders (27.8% versus 27.7%), stroke (2.2% versus 2.5%), and depression (1.1% versus 0.8%). In cohort 1923–24, non-participants and participants were similar regarding sex (women 64.1% versus 62.9%), and National Inpatient Register diagnoses for cardiovascular disorders (35.9% versus 38.9%), stroke (8.3% versus 7.9%), and mental disorders (7.0% versus 4.2%). However, non-participants had higher 3-year mortality rate (23.3% versus 16.6%; *P* = 0.011), and higher prevalence of depression (3.5% versus 1.2%; *P* = 0.022), compared to participants [23].

The Ethics Committee for Medical Research at the University of Gothenburg approved the study and all participants, or a close relative, gave informed consent to participate according to the Declaration of Helsinki [23].

### Examinations

The examinations were conducted at an outpatient clinic or in the participant’s home, and included comprehensive cognitive, psychiatric, social, somatic, and functional examinations [22].

The semi-structured neuropsychiatric examinations, performed by a psychiatrist in 1986–87 and by experienced psychiatric research nurses in 2008–10, included assessments of psychiatric symptoms, signs of dementia, tests of mental functioning (e.g. memory, proverbs, language, visuospatial and executive abilities, apraxia, construction, agnosia), the Mini Mental State Examination (MMSE) [24] and the Alzheimer’s Disease Assessment Scale – cognitive Subscale (ADAS-Cog) [25]. The nurses conducting the examination in 2008–10 were trained and supervised by the principal investigator (IS), who performed the examinations in 1986–87. Inter-rater reliability for signs and symptoms used to diagnose dementia was tested by dual ratings by psychiatric research nurses or psychiatrists. Inter-rater agreement varied between 89.4 and 100.0% (Kappa values 0.74–1.00) [23].

Semi-structured interviews with close informants were performed at baseline in 451 participants (91%) in 1986–87 and 443 (78%) in 2008–2010. The close informant interviews comprised questions about changes in behaviour and intellectual function (e.g. changes in personality, memory, intellectual ability, language, visuospatial function, psychiatric symptoms, activities of daily living), and background factors (e.g. history of stroke/TIA) [23].

### Diagnosis of dementia

The diagnostic procedures were identical at both examinations, as described in detail previously [22]. First, a diagnosis of dementia was made from the psychiatric examination and the close informant interview separately using an algorithm based on the DSM-III-R criteria [26]. A final diagnosis was made from the combined information. All diagnoses were re-evaluated in 2019 to make diagnostic procedures as similar as possible. In this process, one individual born 1923–24 was no longer considered to have dementia.

The details of the classification of different etiological subgroups are described previously [27]. Alzheimer’s disease (AD) was classified according to NINCDS-ADRDA-criteria [28], vascular dementia (VAD) was diagnosed similar to NINDS-AIREN-criteria [29], mixed dementia was diagnosed when there was a history of stroke/TIA without clear temporal connection with dementia onset (more than one year), and other causes were diagnosed when other disorders of sufficient degree to produce dementia evolved in temporal connection with dementia.

### Diagnosis of other diseases

Information about other diseases (i.e. cancer, cerebrovascular disorders, congestive heart failure, diabetes, chronic bronchitis, atrial fibrillation, angina pectoris, and myocardial infarction) was obtained through semi-structured interviews, laboratory tests, information on medications, and the National Inpatient Register. Detailed information on diagnostics are found in online resource 1.

### Other covariates

Educational level was self-reported as compulsory education only or at least one more year.

### Information on mortality

Information on date of death was available from the census records administrated by the Swedish Tax Agency, which records all deaths of Swedish citizens [30].

### Statistical methods

Baseline characteristics were compared using Pearson’s Chi-square test for categorical variables, independent samples t-test for differences in means, and Mann–Whitney U Test for differences in medians. Median survival time and 95% confidence interval (CI) was derived from Kaplan–Meier survival analysis. Cox proportional hazards models were used to compare mortality between the cohorts in five models, presented as Hazard ratios (HR) and 95% confidence intervals (CI). As mean age was slightly higher in cohort 1923–24 [23], all models were adjusted for exact age. Model 1 adjusted for age and sex. Dementia was added in Model 2. Dementia severity was used instead of dementia in Model 3, as cohort 1923–24 had less mild dementia [23]. In Model 4, educational level was added. In Model 5, relevant diseases were added. To select diseases to include in Model 5, primary analyses were performed where each disease was analysed in relation to mortality, using age and sex as covariates and included in Model 5 if the *P*-values in the primary analyses were ≤ 0.2.

Analyses stratified by sex and dementia status were then performed using Models 1–5.

In order to investigate potential effect modification by cohort, the interaction terms (sex*cohort), (dementia status*cohort), and (education*cohort) were used.

Population attributable risk (PAR), which takes into consideration both the relative risk for mortality in individuals with the disease and the prevalence of the disease in the population, was calculated according to the formula:

$${\text{PAR}}\, = \,{\text{p}}\left( {{\text{r}}\, - \,{\text{1}}} \right)/\left[ {{\text{p}} \times {\text{r}}\, + \,\left( {{\text{1}}\, - \,{\text{p}}} \right)} \right].$$

In this formula, p is the proportion of the population with the risk factor and r is the relative risk [31]. The relative risk was derived from the fully adjusted Cox proportional hazards model. When selecting diseases to include in the PAR analyses, the two cohorts were analysed separately.

All models met the proportional hazards assumption based on Schoenfeld residuals. Analyses were performed using IBM SPSS STATISTICS 25 and R Statistical Software (version 3.5.3).

## Results

Characteristics of the samples are given in Table 1, as partly reported previously [23]. Compared to cohort 1901–02, cohort 1923–24 was older, included a higher proportion of men, a higher proportion with more than compulsory education, were more often married, had a distribution of the MMSE towards higher scores, had more often a history of cerebrovascular disorders, congestive heart failure, diabetes mellitus, myocardial infarction, less often a history of atrial fibrillation, and had more often cholesterol and hypertension treatment.

**Table 1:** Characteristics of 85-year-olds in the Gothenburg Birth Cohort studies at baseline

Cohort	1901–02 (n = 494)	1923–24 (n = 571)
Female sex % (n)	71.1 (351/494)	62.9 (359/571)*
Age at baseline, mean (SD)	85.5 (0.1)	85.9 (0.2)***
More than mandatory education % (n)	24.9 (113/454)	56.0 (311/555)***
MMSE, median (IQR) (n)	27 (22–29) (491)	27 (24–29) (555)**
Married, % (n)	23.9 (117/490)	35.5 (195/550)***
Sheltered living, % (n)	24.1 (119/494)	13.3 (76/561)***
Dementia, % (n)	29.8 (147/494)	21.5 (123/571)**
*Dementia severity*		
Mild % (n)	27.9 (41/147)	10.6 (13/123)***
Moderate % (n)	34.7 (51/147)	46.3 (57/123)
Severe % (n)	37.4 (55/147)	43.1 (53/123)
Cancer % (n)	7.3 (36/494)	9.3 (53/571)
Cerebrovascular disorders % (n)	18.8 (93/494)	24.3 (139/571)*
Congestive heart failure % (n)	25.5 (126/494)	32.4 (185/571)*
Diabetes mellitus % (n)	9.1 (45/494)	14.7 (84/571)
Chronic bronchitis % (n)	11.7 (58/494)	8.4 (48/571)
Atrial fibrillation % (n)	18.4 (91/494)	24.9 (142/571)*
Angina pectoris % (n)	21.9 (108/494)	20.0 (114/571)**
Myocardial infarction % (n)	11.7 (58/494)	19.3 (110/571)
Total cholesterol mmol/L, mean (SD), (n)	5.4 (1.2) (425)	5.3 (1.2) (514)
Cholesterol treatment	0.0 (0/494)	20.1 (111/553)***
Hypertension, % (n)	82.4 (406/493)	56.8 (300/528)***
Hypertension treatment, % (n)	17.5 (83/475)	54.9 (298/543)***

Statistical analysis: Pearson’s Chi-square test for categorical variables, independent samples t-test for differences in means, and Mann–Whitney U Test for differences in medians.

*P < 0.05, **P < 0.01, ***P < 0.001, all other values are not significant

### Total mortality

The median survival time from baseline in cohort 1901–02 was 4.9 (95% CI 4.4–5.5) years, and in cohort 1923–24 5.7 (95% CI 5.2–6.2) years. Women had lower mortality than men both in cohort 1901–02 (HR 0.7; 95% CI 0.5–0.8) and cohort 1923–24 (HR 0.8; 95% CI 0.6–0.96).

When adjusting for sex and age (Model 1), mortality was lower in cohort 1923–24 compared to cohort 1901–02 (HR 0.6; 95% CI 0.5–0.7), both in women (HR 0.6; 95% CI 0.4–0.7) and in men (HR 0.6; 95% CI 0.4–0.9). The cohort differences remained in the total group and in women in all models. Among men, the cohort difference was no longer significant when adding education (online resource 2). The interaction terms (sex*cohort), and (education*cohort) were not significant, indicating that cohort changes in mortality did not differ by sex or education.

### Mortality in relation to dementia

Mortality was higher in those with dementia compared to those without, both in cohort 1901–02 (HR 2.6; 95% CI 2.1–3.2), and in cohort 1923–24 (HR 2.9; 95% CI 2.3–3.6), when adjusting for age and sex. Compared to individuals without dementia, mortality was higher in AD (cohort 1901–02, HR 2.2; 95% CI 1.9–3.0, cohort 1923–24, HR 2.6; 95% CI 1.9–3.4), VAD (cohort 1901–02, HR 3.3; 95% CI 2.3–4.7, cohort 1923–24, HR 3.5; 95% CI 2.4–5.2), and in VAD or mixed dementia (cohort 1901–02, HR 3.4; 95% CI 2.5–4.7, cohort 1923–24, HR 3.5; 95% CI 2.5–4.9).

**Fig. 2:**
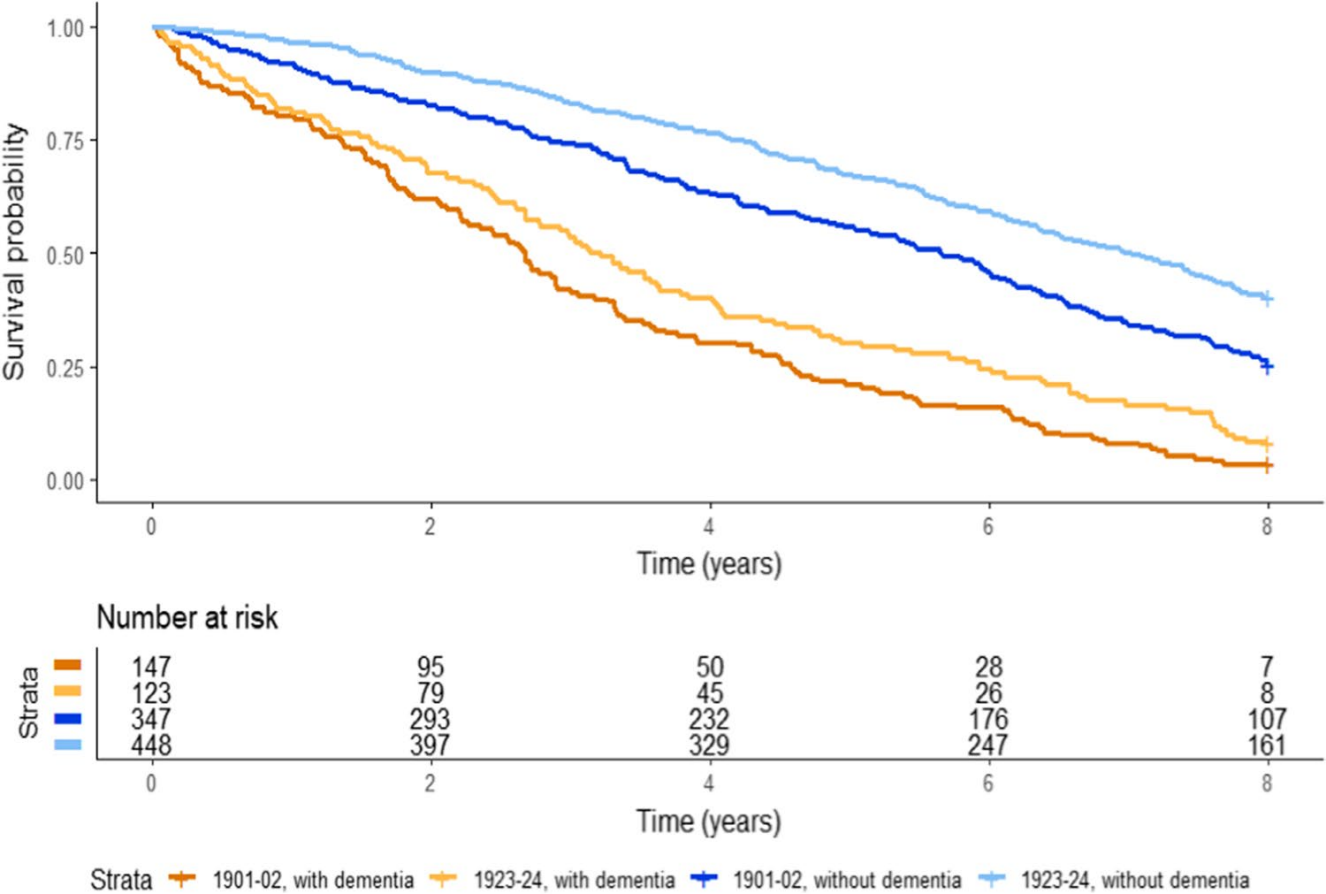
Eight-year survival probability in the two cohorts by dementia status at baseline**.** Kaplan Meier survival analysis was used to analyze the survival. The survival probability was calculated as time from the baseline examination to age at death, or the end of the study period. The analysis is adjusted for sex and exact age at baseline

When stratifying by dementia status, the median survival time from baseline among those without dementia in cohort 1901–02 was 6.0 (95% CI 5.5–6.5) years and in cohort 1923–24 6.5 (95% CI 6.0–7.0) years. Among those with dementia, the median survival time from baseline was 2.7 (95% CI 2.4–3.0) years in cohort 1901–02 and 3.0 (95% CI 2.5–3.5) years in cohort 1923–24. The mortality declined between cohorts among those without dementia (HR 0.7; 95% CI 0.5–0.9), but the decline was not significant among those with dementia (HR 0.7; 95% CI 0.5–1.0) (Model 1) (Fig. 2).

Mortality was related to dementia severity in both cohorts (data not shown). Adding dementia severity to the models, mortality was lower in cohort 1923–24 compared to cohort 1901–02 also among those with dementia (Model 3, online resource 2). In Model 4 (online resource 2), the cohort difference remained in those without dementia, but not in those with. In the fully adjusted model (Model 5, Table 2), mortality was lower both among those without (HR 0.7; 95% CI 0.5–0.9) and with dementia (HR 0.7; 95% CI 0.5–0.99). Similar results were observed for both sexes, but changes only reached statistical significance in dementia-free women (Table 2). The interaction term (dementia status*cohort) was not significant, indicating that cohort changes in mortality did not differ by dementia status.

**Table 2:** Change in 8-year mortality between birth cohorts 1901–02 and 1923–24, stratified by dementia status and sex

	Proportion deceased	Model 1		Model 2		Model 5	
		HR (95%CI)	*P-value*	HR (95%CI)	*P-value*	HR (95%CI)	*P-value*
*Total population*							
Total							
Cohort 1901	76.9	1.0 (Ref.)		1.0 (Ref.)		1.0 (Ref.)	
Cohort 1923	70.4	**0.6 (0.5–0.7)**	** < .001**	**0.7 (0.6–0.8)**	** < .001**	**0.7 (0.5–0.8)**	** < .001**
Women							
Cohort 1901	73.5	1.0 (Ref.)		1.0 (Ref.)		1.0 (Ref.)	
Cohort 1923	66.9	**0.6 (0.4–0.7)**	** < .001**	**0.7 (0.6–0.9)**	**.008**	**0.6 (0.5–0.8)**	**.001**
Men							
Cohort 1901	85.3	1.0 (Ref.)		1.0 (Ref.)		1.0 (Ref.)	
Cohort 1923	76.4	**0.6 (0.4–0.9)**	**.006**	**0.6 (0.4–0.9)**	**.009**	0.8 (0.5–1.1)	.167
*Dementia at baseline*							
Total*							
Cohort 1901	95.2	1.0 (Ref.)		–	–	1.0 (Ref.)	
Cohort 1923	93.5	0.7 (0.5–1.0)	.055	–	–	**0.7 (0.5–0.99)**	**.042**
Women							
Cohort 1901	94.4	1.0 (Ref.)		–	–	1.0 (Ref.)	
Cohort 1923	91.8	0.8 (0.5–1.2)	.219	–	–	0.7 (0.4–1.1)	.095
Men							
Cohort 1901	97.4	1.0 (Ref.)		–	–	1.0 (Ref.)	
Cohort 1923	97.4	0.6 (0.3–1.1)	.103	–	–	0.7 (0.3–1.7)	.463
*Dementia-free at baseline*							
Total*							
Cohort 1901	69.2	1.0 (Ref.)		–	–	1.0 (Ref.)	
Cohort 1923	64.1	**0.7 (0.5–0.9)**	**.002**	–	–	**0.7 (0.5–0.9)**	**.004**
Women							
Cohort 1901	64.2	1.0 (Ref.)		–	–	1.0 (Ref.)	
Cohort 1923	59.1	**0.7 (0.5–0.9)**	**.013**	–	–	**0.6 (0.4–0.8)**	**.003**
Men							
Cohort 1901	80.8	1.0 (Ref.)		–	–	1.0 (Ref.)	
Cohort 1923	71.8	0.7 (0.4–1.01)	.054	–	–	0.7 (0.5–1.1)	.181

Hazards ratios derived from Cox proportional hazards model. All models in the total population are also adjusted for sex. Bolded P-values and hazard ratios have a *P*-value < 0.05.

Model 1: adjusted for age

Model 2: adjusted for age and baseline dementia

Model 3 and 4: See online resource 2

Model 5: adjusted for age, baseline dementia severity (in the dementia group), education, and relevant diseases

### Population attributable risk (PAR) (Table 3)

**Table 3:** Population attributable risk for diseases predicting 8-year mortality, stratified by sex and cohort

Total	% with disease	HR (95% CI)	*P-value*	PAR. %	% with disease	HR (95% CI)	*P-value*	PAR. %
	Cohort 1901-02^a^				Cohort 1923-24^b^			
Dementia	29.8	2.6 (2.0–3.2)	< .001	31.7	21.5	2.8 (2.2–3.5)	< .001	27.7
Cerebrovascular disorders	18.8	1.3 (1.0–1.7)	.043	5.4	24.3	1.4 (1.1–1.7)	.009	8.0
Myocardial infarction	11.7	1.8 (1.3–2.4)	< .001	8.4	19.3	1.3 (1.0–1.8)	.029	6.2
Heart failure	*				32.4	1.3 (1.1–1.7)	.013	9.8
	Cohort 1901-02^c^				Cohort 1923-24^d^			
AD	14.4	2.4 (1.8–3.2)	< .001	16.6	11.6	2.8 (2.1–3.8)	< .001	17.3
VAD	8.7	2.9 (1.9–4.4)	< .001	14.1	5.1	2.2 (1.4–3.4)	.001	5.6
Mixed	3.6	3.7 (2.3–6.2)	< .001	9.1	2.3	2.9 (1.6–5.2)	< .001	4.2
Other	3.0	2.1 (1.2–3.7)	.007	3.3	2.6	4.4 (2.5–7.6)	< .001	8.1
Cerebrovascular disorders	*				24.3	1.5 (1.1–1.9)	.004	10.1
Myocardial infarction	11.7	1.7 (1.3–2.4)	< .001	8.0	19.3	1.4 (1.1–1.8)	.020	6.7
Heart failure	*				32.4	1.3 (1.1–1.7)	.013	9.8
	Cohort 1901-02^e^				Cohort 1923-24^f^			
*Women*								
Dementia	30.8	2.8 (2.1–3.7)	< .001	34.9	23.7	2.6 (2.0–3.5)	< .001	28.0
Cerebrovascular disorders	19.7	1.5 (1.1–2.0)	.014	8.5	22.0	1.4 (1.1–1.9)	.023	8.6
Atrial fibrillation	19.7	1.4 (1.0–2.0)	.034	7.7	*			
Myocardial infarction	10.8	2.3 (1.6–3.4)	< .001	12.6	*			
	Cohort 1901-02^ g^				Cohort 1923-24^ h^			
*Men*								
Dementia	27.3	2.2 (1.5–3.3)	< .001	25.1	17.9	3.3 (2.2–5.0)	< .001	29.6
Chronic bronchitis	16.1	1.9 (1.2–3.1)	.012	12.3	**			
Heart failure	*				27.4	1.5 (1.0–2.1)	.050	11.0
Atrial fibrillation	**				28.8	1.5 (1.1–2.2)	.027	13.4

Cox regressions models to analyse which disorders that predict 8-year mortality. Disorders that remained significant predictors (at *p* < 0.05) in the fully adjusted models are presented in the table. All models are adjusted for exact age

^*^Not significant in the fully adjusted model

^**^Not significantly related to mortality

^a^Adjusted for sex, dementia, cerebrovascular disorders, congestive heart failure, diabetes mellitus, chronic bronchitis, atrial fibrillation, and myocardial infarction

^b^Adjusted for sex, dementia, cerebrovascular disorders, congestive heart failure, diabetes mellitus, chronic bronchitis, atrial fibrillation, angina pectoris, and myocardial infarction

^c^Adjusted for sex, AD, VAD, mixed dementia, other types of dementia, cerebrovascular disorders, congestive heart failure, diabetes mellitus, chronic bronchitis, atrial fibrillation, angina pectoris, and myocardial infarction

^d^Adjusted for sex, AD, VAD, mixed dementia, other types of dementia, cerebrovascular disorders, congestive heart failure, diabetes mellitus, chronic bronchitis, atrial fibrillation, angina pectoris, and myocardial infarction

^e^Adjusted for dementia, cerebrovascular disorders, congestive heart failure, atrial fibrillation, and myocardial infarction

^f^Adjusted for dementia, cerebrovascular disorders, congestive heart failure, diabetes mellitus, chronic bronchitis, atrial fibrillation, angina pectoris, and myocardial infarction

^g^Adjusted for dementia, congestive heart failure, diabetes mellitus, chronic bronchitis, myocardial infarction, and cancer

^h^Adjusted for dementia, cerebrovascular disorders, congestive heart failure, atrial fibrillation, and myocardial infarction

Independent predictors for 8-year mortality in both cohorts were sex, dementia (cohort 1901–02 PAR 31.7%, cohort 1923–24 PAR 27.7%), cerebrovascular disorders (cohort 1901–02 PAR 5.4%, cohort 1923–24 PAR 8.0%), and myocardial infarction (cohort 1901–02 PAR 8.4%, cohort 1923–24 PAR 6.2%). Congestive heart failure was an independent predictor only in cohort 1923–24 (PAR 9.8%).

When adding dementia subgroups instead of total dementia to the model, sex, AD (PAR 1901–02 16.6%, PAR 1923–24 17.3%), VAD (PAR 1901–02 14.1%, PAR 1923–24 5.6%), mixed dementia (PAR 1901–02 9.1%, PAR 1923–24 4.2%), other dementia types (PAR 1901–02 3.3%, PAR 1923–24 8.1%), and myocardial infarction (PAR 1901–02 8.0%, PAR 1923–24 6.7%) were independent predictors for 8-year mortality both in cohort 1901–02 and 1923–24. In cohort 1923–24, cerebrovascular disorders (PAR 10.1%) and heart failure (PAR 9.8%) were also independent predictors for 8-year mortality. Results for subgroups stratified by sex can be seen in online resource 3.

Among women, independent predictors for 8-year mortality in both cohorts were dementia (PAR 1901–02 34.9%, PAR 1923–24 28.0%), and cerebrovascular disorders (PAR 1901–02 8.5%, PAR 1923–24 8.6%). In cohort 1901–02, atrial fibrillation (PAR 7.7%) and myocardial infarction (PAR 12.6%) were also independent risk factors.

Among men, independent predictors for 8-year mortality in both cohorts was dementia (PAR 1901–02 25.1%, PAR 1923–24 29.6%). In cohort 1901–02, chronic bronchitis (PAR 12.3%) was also an independent risk factor. In cohort 1923–24, heart failure (PAR 11.0%) and atrial fibrillation (PAR 13.4%) were also independent predictors.

## Discussion

We aimed to examine whether 8-year mortality in relation to dementia, and whether the importance of dementia to predict mortality relative to other common diseases, changed between two representative samples of 85-year-olds from Gothenburg, Sweden, examined 22 years apart (1986–87 and 2008–10). We found that mortality decreased to a similar extent in those with and without dementia, and that dementia remained the major predictor of death with population attributable risks of 32% 1986–87 and 28% in 2008–10, despite a lower prevalence of dementia in the cohort born 1923–24.

In relation to our first aim, the finding that mortality decreased to a similar extent in individuals with and without dementia is similar to findings from the Kungsholmen Study from central Stockholm, comparing populations aged 75 years and older examined in 1987–89 and 2001–2004 [18]. The French PAQUID study, which compared populations aged 65 years and older examined in 1993 and 2002 [17], found that mortality decreased in both groups, but that the decrease was more pronounced in those without dementia. In contrast, the Framingham study comparing populations aged 60 years and older, examined 1977–1984 and 2004–2008, reported a higher mortality after diagnosis of incident dementia in the later cohort [21]. They suggested that the absolute number of years lived with dementia decreased over the 30-year period. In line with this, the MRC CFAS study from UK reported a compression of cognitive morbidity in women examined in 1991 and 2011, as a result of gain in years without cognitive impairment and a reduction in years lived with cognitive impairment [32]. The reason for discrepancies could be that these latter studies examined mortality in incident dementia, while we examined mortality in prevalent dementia. We have previously reported that the decrease in dementia prevalence was most accentuated for mild dementia [23], suggesting a more compressed disease course in later cohorts. Other explanations for discrepancies include the high age of our sample, different examination years, or differences in diagnostic criteria. More studies are needed on whether mortality decreases differently between persons with and without dementia, as this may have important implications for health care planning and future burden of dementia on society.

Explanations for the increased survival with dementia include a growing awareness that health-care interventions should be provided to patients with dementia [11], including person-centred care [11, 18, 33]. However, this does not explain why mortality decreased to a similar extent in those with and without dementia. Our finding that the excess mortality in those with dementia persisted suggests that it is not the factors affecting increased risk of mortality in dementia that have changed. Instead, the findings suggest that those with and without dementia were affected to a similar extent by factors decreasing mortality over time. The two cohorts are born 22 years apart, and their life-courses have therefore been affected by different societal contexts across historical times [34], including improvements in health care and living conditions. In addition, we have previously reported improvements in health-related factors among later born cohorts, e.g. improved lung function [14], decreased blood pressure [15], and decreased prevalence of cardiovascular disorders [16], which may have had beneficial effects on mortality. In addition, among these cohorts of 85-year olds, the prevalence of hypertension was lower and antihypertensive treatment more common in the later born cohort, while statins were not yet introduced in the 1980s. The Global Burden of Disease Study reported that while the total number of deaths from dementia increased by 40% between 2005 to 2015, an age-standardized decrease was found [35], maybe reflecting a reduced burden of cardiovascular diseases. However, neither dementia nor educational level or any of the selected somatic disorders explained the cohort difference in mortality in our study. Similarly, educational level [18, 21] and vascular factors [17] did not explain the decrease in mortality in previous studies.

In relation to our second aim, we found that dementia remained the major predictor of mortality over more than two decades. Thus, PAR for dementia was substantially higher than for any other diseases in both cohorts, and dementia predicted 32% of deaths among 85-year-olds in the 1980s and 28% of mortality in the 2010s. Similar to our findings for dementia, a MRC CFAS study reported that the relationship between frailty and mortality remained stable across cohorts [36], in contrast to a previous study from our group, where frailty was related to lower mortality in a later-born cohort [37]. Interestingly, the gender gap in PAR for dementia decreased from cohort 1901–02 (25% in men and 35% in women) to cohort 1923–24 (30% in men and 28% in women). Cerebrovascular disorders and myocardial infarction predicted mortality in both cohorts, with PARs ranging from 5–8%. In a previous study on 95-year-olds, PAR for dementia in relation to mortality was 30% in men and 44% in women, suggesting that the effect of dementia on mortality increases with age, at least among women [38]. The prevalence of dementia have a major impact on mortality rates, which has important public health implications [4]. More studies are therefore needed on the relative importance of dementia on overall mortality.

The increased mortality in dementia is well-established [2, 12, 19, 20, 38, 39], but the reasons are not clear. Possible explanations include that brain control systems which are vital for survival, such as regulation of cardiac function, blood pressure, appetite, and energy balance [40, 41] might be affected by the pathologic processes of dementia [38]. Another reason may be that the detection rate of other diseases is lower among individuals with dementia compared to their aged peers [42].

Our results showed that mortality has decreased in both men and women, and we found no interaction between sex and cohort in relation to mortality, indicating no sex difference in the decrease. This is in contrast to the view that decline in mortality has been steeper among men, resulting in a decreased gender gap in life expectancy [43, 44] However, results from previous studies show varying patterns. Mortality among individuals above age 85 in the MRC CFAS study decreased more in women than among men [32], but in the Kungsholmen study mortality decreased similarly in men and women [18]. Among those with dementia, a declining mortality was only observed in women in Kungsholmen and PAQUID [17, 18]. Whether sex differences in risk factors for dementia, such as vascular factors [45] and depression [46] contribute to sex differences in mortality among individuals with dementia remains unclear.

Among the strengths of our study are identical methods and diagnoses in two population-based samples of 85-year-olds examined two decades apart. Further, dementia diagnoses were based on information from both neuropsychiatric examinations and close informant interviews, and all participants were examined for dementia without screening. In addition, we derived date of death from the Swedish Tax agency, which records all deaths of Swedish citizens [30]. There are also some possible limitations. First, the relatively small sample sizes might affect the power of the analyses, especially when stratifying by sex and dementia status. Second, the response rate ranged from 60 to 63%, which is satisfactory in this age group, but we cannot exclude the possibility that response rates among those with dementia have changed, or that characteristics of non-responders differ between cohorts. Third, three-year mortality rate was higher among non-responders in cohort 1923–24, while no such difference was observed in cohort 1901–02. This could have accentuated differences in mortality between cohorts. Fourth, more participants agreed to close informant interviews in cohort 1901–02 compared to 1923–24. It is possible that this led to fewer identified cases of dementia in the latter cohort. Fifth, cohort 1923–24 was slightly older than cohort 1901–02 due to a delayed study start of a few months. The study on the cohort born 1923–24 also took longer time to complete than the study on the cohort born 1901–02 (648 versus 504 days). We have therefore adjusted for exact age in all models. Sixth, information on covariates are based on self-report and the National Inpatient Register, which could have been affected by changes in awareness of diseases. In addition, previous studies have shown that some diseases are under-diagnosed among individuals with dementia [47]. This could potentially affect PAR in our study. In conclusion, we found that mortality among 85-year-olds born 22 years apart declined to a similar degree among those with and without dementia. Dementia remained the most important predictor of death in both cohorts. The relative risk for mortality with dementia did not change between cohorts, despite a decreased mortality rate in the population. The reasons behind the high mortality rate in dementia needs to be further elucidated in future studies.

## Supplementary Information

Below is the link to the electronic supplementary material.

Supplementary file1 (DOCX 24 kb)

## Data Availability

The datasets generated during and analyzed during the current study are available from the corresponding author on reasonable request, and according to regulations of the General Data Protection Regulation.
