# Supplementary material for: Dementia remains the major predictor of death among octogenarians. A study of two population cohorts of 85-year-olds examined 22 years apart
Source: Eur J Epidemiol. 2021 Apr 21;36(5):507–17. doi: 10.1007/s10654-021-00745-5 (PMC8159837; doi:10.1007/s10654-021-00745-5)
Supplement: Supplementary file 1 — Supplementary file1 (DOCX 24 kb) [file 10654_2021_745_MOESM1_ESM.docx]

**List of online only material**

1. Online resource 1. Detailed information on diagnostics of the included diseases

2. Online resource 2. Change in 8-year mortality between birth cohorts 1901-02 and 1923-24, stratified by dementia status and sex, Model 3 and 4

3. Online resource 3. Population attributable risk for diseases predicting 8-year mortality, stratified by sex and cohort

4. References for eTable1.

# Online resource 1. Detailed information on diagnostics of the included diseases

| **Disease** | **Information from interviews** | **Laboratory results** | **Medications** | **the National Inpatient Register^a^** |
| --- | --- | --- | --- | --- |
| Myocardial infarction | self-reported | moderate or major Q-waves on ECG, MC 1-1 and 1-2, except 1-2-6 and 1-2-8 |  | ICD8-SE 410-410.99, 412.01, 412.91  ICD10 I21-I23, I24.1, I25.2, I25.6 |
| Diabetes mellitus | self-reported | having one venous blood glucose value of 11.1 mmol/l or more, or HbA1c of 6.5 or more | ATC codes A10A, A10B | ICD8-SE 250  ICD10-SE E10-E14 |
| Angina pectoris | based on information obtained through interviews and diagnosed according to the Rose criteria [1] |  | ATC code C01DA | ICD8-SE; 413, ICD10-SE; I20.0, I20.8, I20.9 |
| Congestive heart failure | nurses observation or self-reported lower leg oedema, orthopnoea |  |  | ICD8-SE 427.00  ICD10-SE I11.0, I13.0, I13.2, I50 |
| Atrial fibrillation |  | ECG, MC 8.3 |  | ICD8-SE 427  ICD10-SE I44-I49 |
| Cerebrovascular disorders | A history of stroke/TIA was determined based on self- or close informant report only if acute focal symptoms (i.e. hemiparesis or aphasia) were reported [2] |  |  | ICD8-SE 430-438  ICD10-SE I60-I69, G45 |
| Cancer |  |  |  | the Swedish Cancer Register, all cancers |
| Chronic bronchitis | self-reported cough with sputum expectoration for at least 3 months per year during at least 2 years |  |  | ICD8-SE 490-492  ICD10-SE J40- J44 |
| Hypertension |  | systolic blood pressure >140 mmHg and/or diastolic blood pressure >90 mmHg |  |  |
| Hypertension treatment | Self-reported |  |  |  |
| Cholesterol treatment |  |  | ATC-code C10 |  |

^a^ Admissions up to five year prior to the baseline examination dates are included in the analyses, except for stroke/TIA were all admissions are included. The National Inpatient Register has full coverage in the region from 1972 (except for 1976), and full coverage from all public health authorities from 1987.[3]

ECG = Electrocardiography, MC = Minnesota Code, HbA1c = glycated haemoglobin

Online resource 2**.** Change in 8-year mortality between birth cohorts 1901-02 and 1923-24, stratified by dementia status and sex, Model 3 and 4

|  | **Proportion deceased** | **Model 3** |  | **Model 4** |  |
| --- | --- | --- | --- | --- | --- |
|  |  | **HR (95%CI)** | ***P-value*** | **HR (95%CI)** | ***P-value*** |
| **Total population** |  |  |  |  |  |
| **Total** |  |  |  |  |  |
| Cohort 1901 | 76.9 | 1.0 (Ref.) |  | 1.0 (Ref.) |  |
| Cohort 1923 | 70.4 | **0.7 (0.6-0.8)** | **<.001** | **0.7 (0.6-0.9)** | **.006** |
| **Women** |  |  |  |  |  |
| Cohort 1901 | 73.5 | 1.0 (Ref.) |  | 1.0 (Ref.) |  |
| Cohort 1923 | 66.9 | **0.7 (0.6-0.9)** | **.007** | **0.7 (0.6-0.95)** | **.021** |
| **Men** |  |  |  |  |  |
| Cohort 1901 | 85.3 | 1.0 (Ref.) |  | 1.0 (Ref.) |  |
| Cohort 1923 | 76.4 | **0.6 (0.5-0.9)** | **.013** | 0.8 (0.5-1.1) | .192 |
|  |  |  |  |  |  |
| **Dementia at baseline** |  |  |  |  |  |
| **Total*** |  |  |  |  |  |
| Cohort 1901 | 95.2 | 1.0 (Ref.) |  | 1.0 (Ref.) |  |
| Cohort 1923 | 93.5 | **0.7 (0.5-0.96)** | **.025** | 0.7 (0.5-1.0) | .051 |
| **Women** |  |  |  |  |  |
| Cohort 1901 | 94.4 | 1.0 (Ref.) |  |  |  |
| Cohort 1923 | 91.8 | 0.7 (0.5-1.1) | .122 | 0.7 (0.4-1.1) | .082 |
| **Men** |  |  |  |  |  |
| Cohort 1901 | 97.4 | 1.0 (Ref.) |  | 1.0 (Ref.) |  |
| Cohort 1923 | 97.4 | 0.6 (0.4-1.2) | .147 | 0.7 (0.3-1.7) | .460 |
|  |  |  |  |  |  |
| **Dementia-free at baseline** |  |  |  |  |  |
| **Total*** |  |  |  |  |  |
| Cohort 1901 | 69.2 | -- | -- | 1.0 (Ref.) |  |
| Cohort 1923 | 64.1 | -- | -- | **0.7 (0.6-0.96)** | **.026** |
| **Women** |  |  |  |  |  |
| Cohort 1901 | 64.2 | -- | -- | 1.0 (Ref.) |  |
| Cohort 1923 | 59.1 | -- | -- | 0.7 (0.5-1.01) | .056 |
| **Men** |  |  |  |  |  |
| Cohort 1901 | 80.8 | -- | -- | 1.0 (Ref.) |  |
| Cohort 1923 | 71.8 | -- | -- | 0.7 (0.5-1.2) | 0.197 |

Hazards ratios derived from Cox proportional hazards model. All models in the total population are also adjusted for sex.

Model 3: adjusted for age and baseline dementia severity (in the dementia group)

Model 4: adjusted for age, baseline dementia severity (in the dementia group), and education

Online resource 3**.** Population attributable risk for diseases predicting 8-year mortality, stratified by sex and cohort

|  | **Cohort 1901-02^a^** | | | | | **Cohort 1923-24^b^** | | | | |
| --- | --- | --- | --- | --- | --- | --- | --- | --- | --- | --- |
| **Women** | **% with disease** | **HR (95% CI)** | ***P-value*** | **PAR. %** | **% with disease** | | **HR (95% CI)** | ***P-value*** | **PAR. %** |  |
| AD | 15.1 | 2.5 (1.8-3.6) | <.001 | 18.8 | 13.6 | | 3.0 (2.1-4.3) | <.001 | 21.6 |  |
| VAD | 10.0 | 2.5 (1.5-4.1) | <.001 | 13.0 | *** | | | | |  |
| Mixed | 4.3 | 4.1 (2.0-7.3) | <.001 | 11.5 | 3.1 | | 2.4 (1.2-4.5) | 0.010 | 4.0 |  |
| Other | 1.4 | 4.9 (2-12.1) | 0.001 | 5.2 | 2.5 | | 3.0 (1.5-6.2) | 0.003 | 4.8 |  |
| Cerebrovascular disorders | 19.7 | 1.5 (1.0-2.2) | 0.050 | 8.5 | 22.0 | | 1.6 (1.2-2.3) | 0.005 | 12.3 |  |
| Atrial fibrillation | 19.7 | 1.4 (1.0-1.9) | 0.042 | 7.3 | *** | | | | |  |
| Myocardial infarction | 10.8 | 2.2 (1.5-3.2) | <.001 | 11.4 | *** | | | | |  |
|  | **Cohort 1901-02^c^** | | | | | **Cohort 1923-24^d^** | | | | |
| **Men** | **% with disease** | **HR (95% CI)** | ***P-value*** | **PAR. %** | **% with disease** | | **HR (95% CI)** | ***P-value*** | **PAR. %** |  |
| AD | 12.6% | 2.5 (1.4-4.3) | .001 | 15.8 | 8.0 | | 2.4 (1.4-4.2) | .002 | 10.3 |  |
| VAD | 5.6% | 6.0 (2.6-13.8) | <.001 | 21.9 | 6.1 | | 3.3 (1.7-6.3) | <.001 | 12.1 |  |
| Mixed | *** | | | | 0.9 | | 12.7 (2.9-56.3) | .001 | 9.9 |  |
| Other | *** | | | | 2.8 | | 12.6 (5.2-30.8) | <.001 | 24.8 |  |
| Chronic bronchitis | 16.1% | 2.0 (1.2-3.3) | .005 | 14.0 | **** | | | | |  |
| Heart failure | *** | | | | 27.4 | | 1.6 (1.1-2.3) | .015 | 13.7 |  |
| Cancer | 7.0% | 2.3 (1.0-5.0) | .044 | 8.1 | **** | | | | |  |

Cox regressions models to analyse which disorders that predict 8-year mortality. Disorders that remained significant predictors (at p<.05) in the fully adjusted models are presented in the table. All models are adjusted for exact age.

* Not significant in the fully adjusted model

** Not significantly related to mortality ^a^ Adjusted for dementia subtype, cerebrovascular disorders, congestive heart failure, atrial fibrillation, and myocardial infarction

^b^ Adjusted for dementia subtype, cerebrovascular disorders, congestive heart failure, diabetes mellitus, chronic bronchitis, atrial fibrillation, angina pectoris, and myocardial infarction

^c^ Adjusted for dementia subtype, congestive heart failure, diabetes mellitus, chronic bronchitis, myocardial infarction, and cancer

^d^ Adjusted for dementia subtype, cerebrovascular disorders, congestive heart failure, atrial fibrillation, and myocardial infarction

**References**

1. Rose GA. The diagnosis of ischaemic heart pain and intermittent claudication in field surveys. Bull World Health Organ. 1962;27:645-58.

2. Liebetrau M, Steen B, Skoog I. Stroke in 85-Year-Olds. Stroke. 2003;34(11):2617-22. doi:10.1161/01.STR.0000094420.80781.A9.

3. Socialstyrelsen. Täckningsgrad för den somatiska och psykiatriska slutenvården. 2019. <https://www.socialstyrelsen.se/globalassets/sharepoint-dokument/dokument-webb/statistik/tackningsgrad-patientregistret.pdf>.
